# Are workplace health promotion programs effective at improving presenteeism in workers? a systematic review and best evidence synthesis of the literature

**DOI:** 10.1186/1471-2458-11-395

**Published:** 2011-05-26

**Authors:** Carol Cancelliere, J David Cassidy, Carlo Ammendolia, Pierre Côté

**Affiliations:** 1 Master of Public Health Program, Faculty of Graduate Studies, Lakehead University, Thunder Bay, Ontario, Canada; 2 Division of Health Care and Outcomes Research, Toronto Western Research Institute, Toronto Western Hospital, Ontario, Canada; 3 Division of Epidemiology, Dalla Lana School of Public Health, University of Toronto, Ontario, Canada; 4 Department of Health Policy, Management and Evaluation, Faculty of Medicine, University of Toronto, Ontario, Canada; 5 Institute for Work and Health, Toronto, Ontario, Canada; 6 Department of Medicine, Division of Rheumatology, Mount Sinai Hospital, Toronto, Ontario, Canada

## Abstract

**Background:**

*Presenteeism *is highly prevalent and costly to employers. It is defined as being present at work, but limited in some aspect of job performance by a health problem.

Workplace health promotion (WHP) is a common strategy used to enhance on-the-job productivity. The primary objective is to determine if WHP programs are effective in improving presenteeism. The secondary objectives are to identify characteristics of successful programs and potential risk factors for presenteeism.

**Methods:**

The Cochrane Library, Medline, and other electronic databases were searched from 1990 to 2010. Reference lists were examined, key journals were hand-searched and experts were contacted. Included studies were original research that contained data on at least 20 participants (≥ 18 years of age), and examined the impacts of WHP programs implemented at the workplace. The *Effective Public Health Practice Project Tool for Quantitative Studies *was used to rate studies. 'Strong' and 'moderate' studies were abstracted into evidence tables, and a best evidence synthesis was performed. Interventions were deemed successful if they improved the outcome of interest. Their program components were identified, as were possible risk factors contributing to presenteeism.

**Results:**

After 2,032 titles and abstracts were screened, 47 articles were reviewed, and 14 were accepted (4 strong and 10 moderate studies). These studies contained preliminary evidence for a positive effect of some WHP programs. Successful programs offered organizational leadership, health risk screening, individually tailored programs, and a supportive workplace culture. Potential risk factors contributing to presenteeism included being overweight, a poor diet, a lack of exercise, high stress, and poor relations with co-workers and management. Limitations: This review is limited to English publications. A large number of reviewed studies (70%) were inadmissible due to issues of bias, thus limiting the amount of primary evidence. The uncertainties surrounding presenteeism measurement is of significant concern as a source of bias.

**Conclusions:**

The presenteeism literature is young and heterogeneous. There is preliminary evidence that some WHP programs can positively affect presenteeism and that certain risk factors are of importance. Future research would benefit from standard presenteeism metrics and studies conducted across a broad range of workplace settings.

## Background

### Definitions

A healthy and productive workforce is critical for economic success and population health. Illness at the workplace can result in lost productivity, which arises from two sources: *absenteeism *and *presenteeism. Absenteeism *refers to an employee's time away from work due to illness or disability [1]. *Presenteeism *refers to the decrease in productivity in employees whose health problems have not necessarily led to absenteeism and the decrease in productivity for the disabled workers before and after their absence period [2]. It is defined as being present at work, but limited in some aspects of job performance by a health problem, and it is often a hidden cost for employers [3]. It includes time not spent on job tasks and decreased quality of work (e.g. product waste and product defects) [4]. Absenteeism and presenteeism are part of a continuum within which workers likely transition back and forth over time [5].

Health promotion in the workplace is defined as preventing, minimizing and eliminating health hazards, and maintaining and promoting work ability [6]. Worker health and wellness is maintaining a balance of the physical, mental and social ingredients, as well as health habits associated with good physical condition, energy and vitality [6].

### Presenteeism: a relatively new field

Presenteeism emerged as a new business issue in the 1990's [7]. It is becoming a significant challenge to maintain a healthy and productive workforce for developed countries due to an increasing number of people affected by chronic health conditions and an aging workforce that is more likely to be affected by these conditions. Subsequent rising health care costs and an increasing awareness of presenteeism losses are escalating the demand for health promotion programs for working populations [8].

Research on interventions to improve presenteeism is still relatively new compared with other workplace issues such as healthcare costs and absenteeism [9-11]. Most of the literature on presenteeism has investigated its measurement [3].

### Measurement of presenteeism

Currently, there is no universal agreement on the most appropriate method for measuring or monetizing presenteeism [1,12]. It is typically measured as the costs associated with reduced work output, errors on the job, or failure to meet company production standards [13]. It is difficult to value presenteeism economically because past studies use different measures of presenteeism, include different populations of workers and use various methods to assign dollar values to their losses. Nevertheless, it appears that economic costs are considerable [1,14,15].

Several self-report instruments have been developed to measure presenteeism across various types of jobs and organizations [1,16-18]. These are useful especially when it is difficult to obtain objective data regarding particular characteristics of a workplace or profession (e.g. the number of parts manufactured). Evidence of their psychometric properties has been reported in varying degrees [3,5,14,17-23]. Some common tools with good psychometric properties include the *Work Limitations Questionnaire (WLQ) *[20,24], *Work Productivity and Activity Impairment (WPAI) *[25,26], and the *Stanford Presenteeism Scale (SPS) *[20,27]. These tools assess presenteeism with the *assessment of perceived impairment *approach, where employees are asked how much their illnesses hinder them in performing common mental, physical, and interpersonal tasks and in meeting job demands.

### WHP programs

WHP programs vary considerably in size and composition, and they have evolved significantly over the past 30 years [28-30]. However, whether or not programs can improve workplace productivity has yet to be determined. While containing health care-related costs and absenteeism have been important strategies for companies, greater gains may be realized by improving on-the-job productivity and investing in preventive and early intervention services [29,31-36].

The primary objective of our study is to review and scientifically appraise the literature on WHP programs to see if they are effective in improving presenteeism among employees. The secondary objectives are to identify components of successful WHP programs and to identify risk factors for presenteeism.

## Methods

### Literature search

The scientific literature published between 1990 and January, 2010 was systematically searched. Primary sources were the electronic databases of The Cochrane Library, Medline, Embase, CINAHL Plus, NLM Gateway, PsychINFO, Evidence in Health and Social Care, AMED and the Trip Database (Additional file 1). The search strategy was reviewed by a reference librarian. Additionally, the reference lists of all relevant studies were examined, and three of the most relevant journals were hand-searched between January, 2005 and February, 2010 (i.e. *Journal of Occupational and Environmental Medicine*, *Ergonomics*, and *Journal of Industrial Medicine*). A Google search for unpublished literature was conducted in January, 2010 using the search terms *presenteeism, health promotion*, and *workplace*. Government and other relevant websites were scanned. Experts and organizations involved with WHP and presenteeism were contacted (Additional file 2).

### Screening the literature

All citations identified through the search strategy were screened. These included English-language reports, published reports of original research (randomized controlled and controlled trials, cohort, pre-post, and ecological studies), systematic reviews, meta-analyses, conference proceedings, government reports, guidelines, and unpublished gray literature manuscripts. To be included, studies had to be original research that contained data on at least 20 human participants; focused on adults 18 years of age or older; and examined WHP programs including all types of measures aimed at promoting health and wellness, or reducing the risk of ill-health. These could be targeted at behavioural, physiological, organizational or environmental changes. The intervention had to be implemented at the workplace but activities were allowed to occur elsewhere. For example, risk factor screening and education could occur at the workplace, but workers were allowed to use external exercise facilities, or seek additional medical attention. The intervention also had to be described in sufficient detail. Exclusion criteria included studies examining only military personnel; return-to-work studies; narrative, editorial, or clinical reviews; opinion papers, editorials, and letters to the editor; studies where interventions were not implemented at the worksite; studies where productivity outcomes were not measured or specified; studies which grouped productivity outcomes together, such as presenteeism and absenteeism, and the results could not be evaluated specifically for presenteeism changes; and studies that measured productivity only in terms of lost earnings. All relevant systematic reviews and meta-analyses were screened to ensure that primary studies were not missed.

### Critical review of the literature

Relevant primary studies were assessed for methodological quality using a tool developed and tested by the Effective Public Health Practice Project (EPHPP) - *EPHPP Quality Assessment Tool for Quantitative Studies *(Additional file 3) [37,38]. It consists of six criteria: selection bias, allocation bias, control of confounders, blinding of outcome assessors, data collection methods, and withdrawals and dropouts. Reviewers were asked to rate each criterion as 'weak', 'moderate', or 'strong'. A final global rating of each study was subsequently determined. The tool has demonstrated reliability, content and construct validity, and the ability to adapt current methods for systematic literature reviews of effectiveness to questions related to public health. The test-retest reliability of the EPHPP is good (i.e. Cohen's Kappa of 0.74). Content validity was established by having six experts review the questionnaire, and it was independently tested on 10 primary studies by four experts in critical appraisal and community health. Construct validity was shown through comparisons with another highly rated instrument, the Guide to Community Preventive Services [39]. The EPHPP is relatively easy to use and an accompanying dictionary clarifies any questions related to the components. Two reviewers independently performed in-depth reviews for each study. A consensus method was used to solve disagreements about study selection and methodological quality. A third reviewer was consulted if disagreement persisted.

### Data extraction and synthesis/analysis

Studies were considered scientifically admissible if they were rated as moderate or strong, or scientifically inadmissible if they were rated as weak, had fatal biases or other important methodological flaws. Table 1 summarizes the criteria for this rating classification [38]. Information was extracted from each admissible study on: (1) country and workplace, (2) study design, (3) characteristics of participants, (4) inclusion and exclusion criteria, (5) intervention(s) and control(s), (6) outcome measurements and follow up periods, and (7) key findings and limitations (Additional files 4 and 5). The heterogeneity of the populations, interventions and outcome measures made it too difficult to compare the studies. A best evidence synthesis was performed and is based only on the results of the strong and moderate studies [40]. Interventions were deemed successful if they improved the outcome of interest and their program components were subsequently identified. Possible risk factors contributing to presenteeism were identified through the literature review.

**Table 1:** Quality Assessment Components and Ratings for EPHPP Instrument

Components	Strong	Moderate	Weak
Selection bias	Very likely to be representative of target population; greater than 80% participation rate	Somewhat likely to be representative of target population; 60-79% participation rate	All other responses or not stated

Design	Randomized Control Trial, Clinical Control Trial	Cohort analytic, case-control, cohort, or interrupted time series	All other designs or design not stated

Confounders	Controlled for at least 80% of confounders	Controlled for 60-79% of confounders	Confounders not controlled for, or not stated

Blinding	Blinding of outcome assessor & participants to intervention &/or research question	Blinding of either outcome assessor or participants	Outcome assessor & participants are aware of intervention &/or research question

Data collection methods	Tools are valid & reliable	Tools are valid but reliability not described	No evidence of validity or reliability

Withdrawals & dropouts	Follow-up rate of > 80% of participants	Follow-up rate of 60-79% of participants	Follow-up rate of < 60% of participants or withdrawals & dropouts not described

Copyright 2004 by John Wiley and Sons. Adapted with permission of the author.

## Results

**Figure 1:**
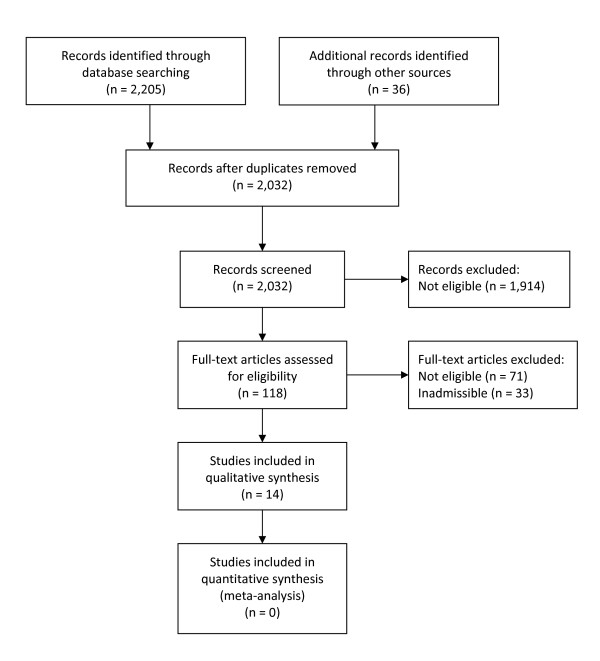
**Flow Diagram of Literature Search**.

After applying the inclusion and exclusion criteria to 2,032 identified titles and abstracts, 47 articles were judged to be relevant and were critically reviewed. Thirty percent of these articles (i.e. 14 unique studies) were deeemed scientifically admissible (Figure 1). Four studies were given a strong rating and 10 studies were rated moderate (Table 1). These studies form the basis of the findings and consist of five randomized controlled trials (RCTs), five cluster RCTs, one interrupted time series study, one crossover designed study, one pre-post study, and one quasi-experimental study (Additional files 4 and 5). The source populations for the studies varied geographically, with three studies from The Netherlands, three from the U.S., two from Japan, two from the U.K., and one each from Canada, Denmark, Finland, and Sweden.

### Interventions demonstrating positive effects on presenteeism

Interventions were deemed successful if they improved the outcome of interest (i.e. presenteeism). Overall, ten interventions demonstrated preliminary evidence of promising effects on presenteeism in their respective employee populations and work settings (Additional files 4, 5 and 6).

Strong evidence for this was found in two studies (Additional files 4 and 6). The first involved worksite exercise [41], and the second investigated the impact of a supervisor education program regarding mental health promotion [42].

The remaining eight studies provided moderate evidence of positive intervention effects (Additional files 5 and 6). These interventions consisted of "A Lifestyle Intervention Via Email" *(Alive!) *[43], extra rest break time for workers engaged in highly repetitive work [44], a multi-disciplinary occupational health program [45], a multi-component health promotion program [46], participatory processes [47,48], exposure to blue-enriched light (vs. white light) [49], and a telephone intervention program for depressed workers [50].

### Interventions not demonstrating improvement in presenteeism

Interventions were deemed unsuccessful if they did not improve the outcome of interest (i.e. presenteeism). Four interventions were unsuccessful at improving presenteeism in their specific employee populations and work settings (Additional files 4 and 5). Two of these were rated as strong [51,52] and two as moderate [53,54]. These consisted of the implementation of a computer mouse with a feedback signal to prevent hovering behaviour [54], a multi-dimensional program for low back pain prevention [51], specific resistance training and all-around physical exercise [53], and worksite exercise/reduced work hours [52].

### Successful vs. unsuccessful interventions

Of the 10 successful interventions, three were delivered by a health professional (Additional files 4, 5 and 6): physiotherapists, occupational health nurses [41], psychologists [42], or occupational health physicians [45]. Two were delivered using a participatory approach, which consists of teamwork among employees, managers, human resources personnel, and researchers [47,48]. One was delivered over the telephone by mental health clinicians [50], one was delivered by email/internet [43], one was an organizational change (i.e. rest break schedule) [44], and one was an environmental change (i.e. lighting) [49]. A final successful intervention utilized a mixed delivery method consisting of email, paper-based packs, and worksite seminars [46]. Of the four unsuccessful interventions (Additional files 4 and 5), one was delivered by a health professional (i.e. physiotherapist) [51], one was delivered by an experienced fitness instructor [53], one was an equipment change (i.e. computer mouse) [54], and the final one consisted of an organizational change (i.e. work hours allocated to mandatory physical exercise) [52].

Some workplaces screened workers prior to intervention. One of the most common screening methods is the health risk assessment (HRA). It includes the assessment of personal health habits and risk factors, estimation of future risk of adverse health outcomes, and feedback in the form of education and counselling to alter risk factors [55]. It is valuable for identifying high-cost claimants; targeting individual program participation and measuring and tracking individual behaviours that effect health care costs, absenteeism and presenteeism [7,55]. Seven of the 10 successful interventions used screening methods (Additional file 6), consisting primarily of HRAs, or other questionnaires/assessments [41,43,45-48,50]. Two of these seven interventions utilized a participatory approach, whereby screening is inherent in the process (i.e. teams first identify problems and needs, then develop solutions) [47,48]. On the other hand, screening methods were used in only one of the four unsuccessful interventions [52].

Seven of the 10 successful interventions tailored their programs to address participant needs (Additional file 6) [41,43,45-48,50], whereas tailoring occurred in only half of the unsuccessful interventions [51,53]. Finally, only one of the 10 successful interventions used an incentive (i.e. lottery tickets) (Additional file 6) [46]. Incentives were not used in any of the unsuccessful interventions.

### Summary of results of strong vs. moderate studies

Four of the 14 admissible studies were rated as strong (Additional file 4). However, there still existed some bias with respect to thorough reporting of withdrawals and drop-outs [51], data collection methods [42], and participant selection [52]. All four studies also showed a risk of bias with respect to blinding of outcome assessors and/or participants, as well as intervention integrity [41,42,51,52].

Three of these four studies were cluster RCTs and the other was a RCT. Two of the four interventions improved presenteeism (Additional files 4 and 6) and involved worksite exercises [41] and a supervisor mental health education program [42]. Both were delivered by health professionals. Workers were screened and the intervention was individually tailored in only one of them [41]. Employee incentives were not used in either intervention.

In two of the four strong studies, the interventions did not improve presenteeism (Additional file 4). These included a low back pain prevention program [51], and a worksite exercise/reduced work hours program [52]. Only one of these was individually tailored and delivered by a health professional [51]. Neither intervention involved worker screening, or the use of incentives.

Of the 14 admissible studies, 10 were rated as moderate evidence and demonstrated similar biases although to a greater degree than the four higher quality studies (Additional file 5). These consist of four RCTs, two cluster RCTs, one crossover design, one interrupted time series, one pre-post study, and one quasi-experimental study. Eight of these 10 interventions improved presenteeism (Additional files 5 and 6). These include Alive (A Lifestyle Intervention Via Email) [43], extra rest break time [44], two participatory interventions [47,48], lighting changes [49], a multi-component health promotion program [46], a telephone support program [50], and an occupational health program [45]. Delivery methods varied and were executed by: health professionals [45,50], participatory methods [47,48], the internet [43], mixed methods [46], an organizational change [44], and an environmental change [49]. Six of these eight successful interventions involved employee screening methods [43,45-48,50]. These six, in addition to the one by Blangsted and colleagues [53], were individually tailored. Only one of the eight interventions used an employee incentive [46].

Two of the 10 moderate studies were not successful at improving presenteeism (Additional file 5) and consisted of exercise [53], and a computer mouse with a feedback signal to prevent hovering behaviour [54]. Neither of these were delivered by health professionals. One was delivered by a fitness intructor [53], and the other involved an equipment change [54]. Only one intervention was individually tailored [53]. Workers were not screened and incentives were not used in either intervention.

A secondary objective was to identify components of WHP programs successful at improving presenteeism. We found preliminary evidence to support the use of one or more of the following: involving employees' supervisors/managers in WHP programs [42,45,47,48], targeting organizational and/or environmental factors to influence behaviour [41,42,44,45,47-49], screening workers prior to intervention using HRAs or other methods [43,45-48,50], improving supervisor/manager knowledge regarding mental health in the workplace [42], allowing physical exercise to occur during working hours [41], and individually tailoring programs [41,43,45-48,50]. Grounding interventions in behaviour change models to help reinforce desirable lifestyle behaviours [43], using participatory approaches with high employee involvement to develop interventions [47,48], and increasing the frequency and duration of rest breaks for workers required to stand for prolonged periods [44] were also helpful. Interventions can be delivered by various modes including telephone, email/internet, seminars, paper-based literature [43,46]; participatory teams [47,48]; organizational changes [44]; environmental changes [49]; and by various health professionals [41,42,45,50].

Another secondary objective was to understand risk factors affecting presenteeism. Several factors were reported in the literature such as being overweight, having a poor diet, smoking, a lack of physical exercise, high stress, poor relations with co-workers and management, and poor physical work environments [1-3]. Health conditions affecting worker presenteeism were also reported and included arthritis, allergies, migraine, chronic pain, diabetes, hypertension, gastro-intestinal conditions, gastro-esophageal reflux disease, musculoskeletal disorders, respiratory disorders, mental health problems such as depression and anxiety, cancer, cardiovascular disease, and metabolic syndrome [1-3,12,13,15]. In general, these risk factors and health conditions can decrease worker productivity by causing pain and fatigue, and by reducing physical and mental capacities.

## Discussion

This systematic review is an important contribution to the field of WHP and presenteeism and builds on previous research in this area. Two previous studies systematically reviewed related literature. Riedel and colleagues [9] searched all English language primary studies, reviews, concept articles, and background articles related to WHP and its effect on worker productivity from 1993 through 1998. They reviewed 146 articles, but found that when productivity loss information was available, it was mostly measured in terms of absenteeism rather than presenteeism. They identified depression screening, back pain exercise programs, smoking cessation, influenza vaccination, and care-seeking programs for minor illnesses as interventions that could provide short term gains in productivity. They concluded that two major challenges to the success of WHP programs were getting high participation rates and maintaining behaviour change over time.

Kuoppala and colleagues [10] studied the association between WHP and job well-being, work ability, absenteeism, and early retirement. Work ability was defined as employees' physical, psychological, and social capacity to work and depends both on their health and the contents of their work. They systematically searched and critically evaluated the literature in Medline and PsychINFO from 1970 to 2005. Out of 1312 references, 46 original studies and systematic reviews were included in their analysis. They found moderate evidence that WHP involving exercise increases work ability. However this finding was based on a study with a weak methodological design and potential biases in participant selection, confounding, blinding, and withdrawals and dropouts [11].

Our findings draw on a larger pool of relevant studies compared to Riedel and colleagues [9]. The review by Kuoppala and colleagues [10] ended in 2005, and involved a broader search including the link between WHP and well-being, work ability, absenteeism, as well as early retirement. Our study updates this search and narrows the focus to analyze only studies investigating the effects of WHP on one or more aspects of presenteeism/worker productivity. Compared to the work of Kuoppala and colleagues [10], our review included more search terms in addition to 'work ability', in an attempt to capture as many presenteeism studies as available (e.g. 'productivity', 'work limitation', 'work impairment', 'presenteeism', 'work performance', and 'work disability') (Additional file 1). We also searched additional databases and the gray literature, and contacted experts in the presenteeism field (Additional file 2).

Consistent with findings from both of the above studies [9,10], our results show that exercise is beneficial in improving presenteeism. Although it is not known which specific type of exercise program is best, if any. Riedel and colleagues found evidence to support back pain exercise programs [9]. Kuoppala and associates identified support for supervised worksite exercise, consisting of aerobic and muscular fitness (one hour, twice per week for nine months). We found evidence to support self-directed worksite exercise (one hour per week for nine months) [41]. Addressing depression and mental health at the workplace was found to be useful in our review, in line with findings by Riedel and colleagues [9]. Again, the specifics of the interventions varied and consisted of depression screening [9], an educational mental health promotion program for supervisors [42], and a depression outreach-treatment telephone program [50].

Our findings suggest, and others agree [10,33] that WHP should target psychosocial factors in addition to physical factors at work. Indeed, the participatory interventions analyzed in our review that addressed both psychosocial and physical factors were beneficial [47,48]. Creating a positive work environment can help to reduce health risks and improve productivity in the workplace.

Our review adds to the existing literature and is useful to employers, health care providers and policy makers. We found additional evidence to support lifestyle behaviour interventions, occupational/multi-component health programs, participatory programs, lighting changes and extra rest break time. We also found other program components that have positive effects on presenteeism such as screening workers prior to intervention, as well as providing workers with incentives and tailored interventions.

In the studies we reviewed, the original investigators described similar ways in which improvements in presenteeism may have been (further) realized in their respective studies. WHP interventions should provide incentives to employees (e.g. monetary) to improve participation and response rates as well as intervention adherence [43]. Interventions such as exercise programs should be longer, more intense and frequent [41,47,53]; and they should be based on a theory such as the behaviour change model [43]. Longer follow-up periods are needed to determine whether intervention effects improve, or persist over the long term [42,46-48,52]. Care must be taken to ensure that interventions are appropriate for specific employee populations and work tasks. For example, individually tailored interventions may not be appropriate in team-based job tasks [51]. Additional resources such as more support, training or counselling sessions, may be required to make interventions more robust [41,51,53]. Moreover, broader actions may be needed such as organizational and environmental interventions that include a multi-professional approach [41,45,47].

Presenteeism and absenteeism are often inter-related. Individuals may only be absent below a certain threshold of illness and quality of life. This threshold may depend on the working situation (e.g. manual or white collar jobs), the type of illness (e.g. mental or physical), the degree of coping and the support available in the worker's social network [3]. Thus, the continuum between absenteeism and presenteeism can vary highly over time. Furthermore, an intervention might be successful in reducing work absence, but only at the expense of a rise in presenteeism, if the health problem is not properly dealt with [56].

Preliminary yet promising effects were found for various WHP programs. However, it is important to mention that some of these program components were also present in the four studies that did not demonstrate improved presenteeism (Additional files 4 and 5). These include physical activity [52,53], ergonomic changes [54] and a multi-dimensional prevention program [51]. Therefore, a distinction needs to be made between *theory failure *and *program failure *[57]. In these four unsuccessful interventions, it is likely that program failure occurred (e.g. the intervention was not implemented properly, or compliance was poor). Theory failure would imply that physical activity, ergonomic changes, or other prevention programs are not effective. However, we did find some of these components to be effective in our review (Additional file 6).

### Review strengths and limitations

This review has several strengths. The best available evidence was systematically gathered, appraised, and synthesized. Limiting the findings to studies of better methodological quality is a notable strength that builds on past results [9,10]. All steps of our comprehensive review were explicitly reported, thereby making the process more transparent and reproducible. Our findings are reported in compliance with PRISMA guidelines (Preferred Reporting Items for Systematic Reviews and Meta-Analysis) [58].

Our study has limitations. While the search was extensive, it is limited to the literature available as of January, 2010 and to English publications. Only 14 of 47 articles reviewed were scientifically admissible, which limits the number of higher quality studies to draw inferences from. The other studies were inadmissible because of bias. Included studies also had risk of bias, but to a lesser extent. Common problems encountered included selection bias, or the selection criteria to get into the study were not described [59-81]. Many of the inadmissible studies had poor response and participation rates (i.e. less than 60%) [59-61,67,69,72,77,82]. Other studies did not consider, or report on, common confounding factors such as gender, age, education, and health status [59,60,62-64,66-71,74-79,82-90]. Insufficient blinding of the assessors and/or the participants was observed in several studies [59-61,63,64,69,70,72,75,79-81,83-85,88-91]. Information bias is likely in a number of the studies as some of the data collection tools have not been shown to be reliable and valid [59,61,63-65,72,78-82,85,87-90]. Finally, various studies did not adequately report withdrawal and/or drop-out rates, or had unacceptably high rates (i.e. follow-up rate less than 60%) with little to no examination of differences between study participants and nonparticipants [59-62,64,65,68-71,73-75,82,83,88,89].

Presenteeism is an evolving field and is difficult to measure. The uncertainties and inconsistencies surrounding its measurement may be one of the biggest limitations regarding the usefulness of this review. All of the current instruments have drawbacks and several significant measurement issues have been raised [1,3,13,14,18,92]; Edington DW, personal communication, March 2010]. Only a few of the instruments have been validated against objective measures of productivity, such as the number of calls made in a call centre, or the number of parts assembled in a manufacturing plant. This is easiest to do when dealing with piecework, but is much more difficult when concrete, measureable output is not available, as with jobs in the knowledge sector, or when dealing with team work where the presenteeism of a single team member may affect the job performance of the group. Additionally, each instrument measures a different quality of presenteeism (e.g. quality or quantity of work), making results difficult to compare. Finally, indirect costs faced by employers having to respond to reduced productivity need to be taken into account, including hiring new or temporary staff, training staff, paying overtime to other employees, and dealing with lost revenue, to name a few.

## Conclusions

WHP represents one of the most significant strategies for enhancing the productivity of workers at a time when their average age is increasing [93]. Despite longstanding advocacy for comprehensive worksite programs, we need more empirical evidence to link these strategies to improvements in health and productivity [35]. We found preliminary evidence of a positive effect for some programs, identified their components and some contributing risk factors for presenteeism. Caution is needed in interpreting these results due to heterogeneous response/participation rates, interventions, intervention delivery methods, presenteeism measurement tools, employee populations, geographical and workplace settings, and inclusion and exclusion criteria. Interestingly, it has been stated that the most important issue for organizations to address is not whether or not WHP programs should be implemented to reduce risks and enhance productivity, but rather how such programs should be designed, implemented, and evaluated to achieve optimal results [47]. Further implementation research is needed in this area.

## Competing interests

The authors declare that they have no competing interests.

## Authors' contributions

All authors formulated the research question, sketched the research design and critically analyzed relevant studies. JDC supervised and coordinated the study. CC reviewed the existing literature, performed the literature search, and drafted the manuscript. CC and CA analyzed the majority of the studies. JDC and PC revised the manuscript substantially. All authors read and approved the final manuscript.

## Pre-publication history

The pre-publication history for this paper can be accessed here:

http://www.biomedcentral.com/1471-2458/11/395/prepub

## Supplementary Material

Additional file 1 **Search Strategy**. This file provides the complete list of search terms used to search The Cochrane Library, Medline, Embase, and other databases. Click here for file

Additional file 2 **Experts, Organizations, & Websites Contacted**. This file provides a list of experts, organizations, and websites contacted that deal with presenteeism and/or workplace health promotion. Click here for file

Additional file 3 **Quality Assessment Tool for Quantitative Studies**. This file contains the Effective Public Health Practice Project's (EPHPP) tool used to assess the methodological quality of studies that passed relevance screening for inclusion in this review. Click here for file

Additional file 4 **Data Extraction Results for Included Studies Rated Strong**. This file contains the data extraction results for the 4 studies included in this review that were rated as strong after being assessed for methodological quality. Data includes authors, date of publication, country, study design, setting, participants, interventions, outcome measurements, and key findings and limitations. Click here for file

Additional file 5 **Data Extraction Results for Included Studies Rated Moderate**. This file contains the data extraction results for the 10 studies included in this review that were rated as moderate after being assessed for methodological quality. Data includes authors, date of publication, country, study design, setting, participants, interventions, outcome measurements, and key findings and limitations. Click here for file

Additional file 6 **Description of Interventions Demonstrating Positive Effect on Presenteeism**. This file contains detailed descriptions of the 10 interventions which demonstrated a positive effect on presenteeism. Data includes intervention goals, intervention delivery methods; and indicates whether subjects were screened, whether interventions were individually tailored, and whether incentives were used. Click here for file
