# Supplementary material for: Are workplace health promotion programs effective at improving presenteeism in workers? a systematic review and best evidence synthesis of the literature
Source: BMC Public Health. 2011 May 26;11:395. doi: 10.1186/1471-2458-11-395 (PMC3123596; doi:10.1186/1471-2458-11-395)
Supplement: Additional file 1 — Search Strategy. This file provides the complete list of search terms used to search The Cochrane Library, Medline, Embase, and other databases. [file 1471-2458-11-395-S1.PDF]

Additional File 1. Search Strategy

COCHRANE LIBRARY search strategy

ID	Search
#1	MeSH descriptor Workplace explode all trees
#2	(#1 OR worksite* OR employee* OR worker* OR corporat* OR employer* OR company*)
#3	MeSH descriptor Occupational Health explode all trees
#4	MeSH descriptor Occupational Health Services explode all trees
#5	MeSH descriptor Health Promotion explode all trees
#6	MeSH descriptor Primary Prevention explode all trees
#7	MeSH descriptor Employee Incentive Plans explode all trees
#8	MeSH descriptor Preventive Health Services explode all trees
#9	(#3 OR #4 OR #5 OR #6 OR #7 OR #8 OR program* OR “wellness program” OR intervention* OR prevent*)
#10	MeSH descriptor Employee Performance Appraisal explode all trees
#11	MeSH descriptor Task Performance and Analysis explode all trees
#12	MeSH descriptor Work Capacity Evaluation explode all trees
#13	MeSH descriptor Efficiency explode all trees
#14	MeSH descriptor Efficiency, Organizational explode all trees
#15	MeSH descriptor Disability Evaluation explode all trees
#16	(#10 OR #11 OR #12 OR #13 OR #14 OR #15 OR presenteeism OR “work* limitation*” OR productivity OR “job performance” OR “work* performance” OR “productivity loss” OR disabilit* OR “work* impairment*” OR “impaired performance”)
#17	(#2 AND #9 AND #16)

MEDLINE search strategy

("Workplace"[Mesh] OR worksite* OR employee* OR worker* OR corporat* OR employer* OR compan*) AND ((((("Occupational Health"[Mesh] OR "Occupational Health Services"[Mesh])) OR "Health Promotion"[Mesh]) OR "Managed Care Programs"[Mesh]) OR ("prevention and control "[Subheading] OR "Primary Prevention"[Mesh]) OR "health program*" OR "wellness program*" OR prevention) AND ("Work Capacity Evaluation"[Mesh] OR ("Efficiency"[Mesh] OR "Efficiency, Organizational"[Mesh]) OR presenteeism OR "work* limitation*" OR "work* productivity" OR "job performance" OR "work* performance" OR "productivity loss" OR "work* disabilit*" OR "disability prevention" OR "work* impairment*" OR "impaired performance") AND (((((((("Review "[Publication Type] OR "Review Literature as Topic"[Mesh] OR "Peer Review, Research"[Mesh])) OR ("Meta-Analysis "[Publication Type] OR "Meta-Analysis as Topic"[Mesh])) OR ("Randomized Controlled Trial "[Publication Type] OR "Randomized Controlled Trials as Topic"[Mesh] OR "Controlled Clinical Trial "[Publication Type])) OR "Multicenter Studies as Topic"[Mesh]) OR "Cohort Studies"[Mesh]) OR "Controlled Clinical Trials as Topic"[Mesh]) OR ("Evaluation Studies "[Publication Type] OR "Evaluation Studies as Topic"[Mesh]) OR ecological OR pre-post)

EMBASE search strategy

ID	Search
#1	'employee'/exp OR 'workplace'/exp OR 'worker'/exp OR 'employer'/exp OR worksite* OR corporat* OR compan*
#2	'occupational health'/exp OR 'occupational health service'/exp OR 'preventive medicine'/exp OR 'health promotion'/exp OR 'prevention and control'/exp OR 'education program'/exp OR 'health program'/exp OR wellness OR intervention OR program
#3	'work capacity'/exp OR 'productivity'/exp OR 'personnel management'/exp OR 'task performance'/exp OR 'job performance'/exp OR 'work disability'/exp OR efficien* OR 'employee performance' OR presenteeism OR 'work limitation' OR 'work performance' OR 'productivity loss' OR 'disability prevention' OR 'work impairment' OR 'impaired performance'
#4	'systematic review'/exp OR 'meta analysis'/exp OR 'randomized controlled trial'/exp OR 'cohort analysis'/exp OR ecological OR 'pre post'
#5	(#1 AND #2 AND #3 AND #4)

CINAHL PLUS search strategy

ID	Search
S1	(MH "Work Environment+")
S2	(MH "Corporations+")
S3	(MH "Workforce")
S4	S1 or S2 or S3
S5	S1 or S2 or S3 or workplace* or worksite* or worker* or employee* or employer* or compan*
S6	(MH "Occupational Health Services+")
S7	(MH "Employee Assistance Programs")
S8	(MH "Employee Incentive Programs")
S9	(MH "Occupational Health+")
S10	(MH "Health Services+")
S11	(MH "Health Promotion+")
S12	(MH "Preventive Health Care+")
S13	(MH "Diet Therapy+")
S14	(MH "Disability Management")
S15	(MH "Health Education+")
S16	(MH "Stress Management")
S17	(MH "Smoking Cessation Programs")
S18	(MH "Weight Reduction Programs")
S19	(MH "Health Screening+")
S20	(MH "Alternative Therapies+")
S21	S6 or S7 or S8 or S9 or S10 or S11 or S12 or S13 or S14 or S15 or S16 or S17 or S18 or S19 or S20
S22	S6 or S7 or S8 or S9 or S10 or S11 or S12 or S13 or S14 or S15 or S16 or S17 or S18 or S19 or S20 or "wellness program*" or prevention
S23	(MH "Employee Performance Appraisal+")
S24	(MH "Job Performance")
S25	(MH "Productivity")
S26	S23 or S24 or S25
S27	S23 or S24 or S25 or presenteeism or "work* limitation*" or "work* productivity" or "work* performance" or "productivity loss" or "work* disabilit*" or "disability prevention" or "work* impairment*" or "impaired performance"
S28	(MH "Systematic Review") or (MH "Cochrane Library")
S29	(MH "Meta Analysis")
S30	(MH "Clinical Trials+")
S31	(MH "Concurrent Prospective Studies") or (MH "Prospective Studies+") or (MH "Nonconcurrent Prospective Studies")
S32	S28 or S29 or S30 or S31
S33	S28 or S29 or S30 or S31 or cohort or ecological or pre-post
S34	S5 and S22 and S27 and S33

NLM GATEWAY search strategy

ID	Search
#1	Workplace[MESH] OR worksite OR employee OR worker OR Corporate OR company OR corporation Limit: English, 1990:2010
#2	Occupational Health[MESH] OR Occupational Health Services[MESH] OR Health Promotion[MESH] OR prevention & control[SH] OR Primary Prevention MESH] OR Employee Incentive Plans[MESH] OR Managed Care Programs[MESH] OR “health program” OR “wellness program” Limit: English, 1990:2010
#3	Employee Performance Appraisal[MESH] OR Work Capacity Evaluation[MESH] OR Efficiency[MESH] OR presenteeism OR “work limitation” OR productivity OR Task Performance and Analysis [MESH] OR “job performance” OR “work performance” OR “productivity loss” OR Disability Evaluation[MESH] OR “work disability” OR “disability prevention” OR “work impairment” OR “impaired performance” Limit: English, 1990:2010
#4	Review Literature as Topic[MESH] OR Meta-Analysis as Topic[MESH] OR Controlled Clinical Trials as Topic[MESH] OR Cohort Studies [MESH] OR ecological OR pre-post OR “systematic review”
#5	#1 AND #2 AND #3 AND #4

EVIDENCE IN HEALTH AND SOCIAL CARE search strategy

(workplace* OR worksite* OR employee* OR worker* OR corporat* OR compan*) AND ("occupational health" OR "occupational health services" OR "health promotion" OR prevention OR program OR "wellness program" OR intervention) AND (presenteeism OR "work* limitation* OR impair* OR "work* productivity" OR efficiency OR "job performance" OR "work* performance" OR "productivity loss" OR "work* disability" OR "disability prevention" OR "work* impairment*" OR "impaired performance") AND ("systematic review" OR "meta analysis" OR "randomized controlled clinical trial" OR cohort OR ecological OR pre-post)

PsychINFO search strategy

1. exp Personnel/
2. exp Business Organizations/
3. (workplace* or worksite* or employee* or worker* or corporat* or employer* or compan*).mp. [mp=title, abstract, heading word, table of contents, key concepts]
4. 1 or 2 or 3
5. exp "Industrial and Organizational Psychology"/ or exp Health Promotion/
6. exp Prevention/ or exp Professional Consultation/ or exp Public Health Services/
7. exp Health Education/
8. exp Educational Programs/ or exp Nutrition/ or exp Physical Education/
9. exp Preventive Medicine/
10. exp Mental Health Programs/
11. exp Well Being/ or exp Employee Assistance Programs/ or exp Holistic Health/ or exp Occupational Therapy/
12. ("occupational health" or wellness or program or intervention).mp. [mp=title, abstract, heading word, table of contents, key concepts]
13. 5 or 6 or 7 or 8 or 9 or 10 or 11 or 12
14. exp Personnel Evaluation/ or exp Job Performance/ or exp Employee Productivity/ or exp Employee Efficiency/
15. exp Disability Evaluation/ or exp Ability Level/
16. exp Monetary Incentives/ or exp Incentives/
17. 13 or 16
18. 14 or 15
19. (presenteeism or "work* limitation*" or "productivity loss" or "work* disabilit*" or "disability prevention" or "work* impairment*").mp. [mp=title, abstract, heading word, table of contents, key concepts]
20. 18 or 19
21. exp Treatment Effectiveness Evaluation/ or exp "Literature Review"/
22. exp Meta Analysis/
23. exp Cohort Analysis/
24. ("randomized controlled trial*" or ecological or pre-post).mp. [mp=title, abstract, heading word, table of contents, key concepts]
25. systematic review.mp. [mp=title, abstract, heading word, table of contents, key concepts]
26. 21 or 22 or 23 or 24 or 25
27. 4 and 17 and 20 and 26

AMED search strategy

ID	Search
S1	(DE "WORKPLACE")
S2	(DE "EMPLOYERS")
S3	S1 or S2
S4	S1 or S2 or worksite* or employee* or worker* or corporat* or compan*
S5	(DE "OCCUPATIONAL HEALTH")
S6	(DE "OCCUPATIONAL HEALTH SERVICES")
S7	(DE "HEALTH PROMOTION") OR (DE "HEALTH SERVICES")
S8	(DE "PREVENTION") OR (DE "PREVENTIVE HEALTH CARE") OR (DE "PREVENTIVE HEALTH SERVICES")
S9	S5 or S6 or S7 or S8
S10	S5 or S6 or S7 or S8 or "health program*" or "wellness program*"
S11	(DE "WORK CAPACITY EVALUATION")
S12	(DE "EFFICIENCY")
S13	(DE "DISABILITYEVALUATION")
S14	S11 or S12 or S13
S15	S11 or S12 or S13 or presenteeism or "work* limitation*" or "work* productivity" or "job performance" or "work* performance" or "productivity loss" or "work* disability" or "work* impairment*" or "impaired performance"
S16	(DE "META ANALYSIS")
S17	(DE "RANDOMIZED CONTROLLED TRIALS")
S18	S16 or S17
S19	S16 or S17 or "systematic review*" or cohort or prospective or evaluation or ecological or pre-post
S20	S4 and S10 and S15 and S19
