# Supplementary material for: Are workplace health promotion programs effective at improving presenteeism in workers? a systematic review and best evidence synthesis of the literature
Source: BMC Public Health. 2011 May 26;11:395. doi: 10.1186/1471-2458-11-395 (PMC3123596; doi:10.1186/1471-2458-11-395)
Supplement: Additional file 2 — Experts, Organizations, & Websites Contacted. This file provides a list of experts, organizations, and websites contacted that deal with presenteeism and/or workplace health promotion. [file 1471-2458-11-395-S2.DOCX]

## **Additional File 2. Experts, Organizations, and Websites Contacted**

American Psychological Association

Association of Workers Compensation Board of Canada (AWCBC)

Balfanz, D., Stanford Health Promotion Network, U.S.

Boocock, M. G., Auckland University of Technology, New Zealand

Buck Consultants, U.S.

Buffett & Company Worksite Wellness Inc., Canada

Canadian Association of Administration of Labour Legislation (CAALL)

Canadian Centre for Occupational Health & Safety (CCOHS)

Canadian Council for Health & Active Living at Work (CCHALW)

Canada/European Union Cooperation on Workplace Safety & Health

Canadian Institute for Health Information

Canadian Labour & Business Centre

Canadian National (CN)

Chevron Corporation, U.S.

Corbiere, M., Sherbrooke University, Canada

Crossman, D., Public Health Agency of Canada

de Boer, A. G. E. M., Coronel Institute for Occupational & Environmental Health, The Netherlands

Diversity Wellness, U.S.

Edington, D. W., University of Michigan, U.S.

European Network for Workplace Health Promotion

Faculty of Public Health, U.K.

Goetzel, R., Cornell University, U.S.

Gold-Knecht Associates, U.S.

The Graham Lowe Group, Inc., Canada

Hall, A., University of Windsor, Canada

Health Canada Workplace Health Bureau

Healthscore Inc., Canada

Health Work & Wellness Group, Canada

Human Resources & Social Development Canada – Labour Program

Human Solutions, Canada

Institute for Corporate Productivity, U.S.

Institute for Work & Health, Canada

Integrated Benefits Institute & National Business Coalition, U.S.

International Commission on Occupational Health (ICOH)

International Stress Management Association, U.K.

J.J. Keller & Associates Inc., U.S.

Jones, E., CDC Occupational Health & Prevention Services, U.S.

Kenny, G. P., University of Ottawa, Canada

Kronos Optimal Health Company, U.S.

Lainez, J. A., Hospital Clinico Universitario, Universidad de Valencia, Spain

Lamminpӓӓ, A., Division of Insurance, Finland

Lerner, D., Tufts-New England Medical Center, U.S.

LewChuk, W., McMaster University, Canada

Loeppke, R., Matria Healthcare, U.S

ManuLife Financial, Canada

The Matheson System, U.S.

McClone Wellness Group, U.S.

MDS Nordion, Canada

Medisys., Canada

Merck Frosst Canada & Co.

Meunier, Y. A., Stanford Health Promotion Network, U.S.

Meverden, A., Microsoft Corporation, U.S.

The National Institute for Occupational Health & Safety (NIOSH), U.S.

National Quality Institute (NQI), Canada

Occupational Health Clinics for Ontario Workers Inc., Canada

Ontario Healthy Workplace Coalition, Canada

Ontario Ministry of Labour, Canada

Owens, A., Qualcomm Inc., U.S.

Pelletier, K. R., University of California School of Medicine, U.S.

Pransky, G., Liberty Mutual, U.S.

Public Health Agency of Canada

Renfrew Insurance, Canada

Rothstein, M., University of Louisville School of Medicine, U.S.

Ruotsalainen, J. H., Finnish Institute of Occupational Health, Finland

Shepell-fgi, Canada

Tompa, E., Institute for Work and Health, Canada

University of Toronto, Centre for Health Promotion, Canada

Verbeek, J., Finnish Institute of Occupational Health, Finland

Veterans Affairs Canada

Vetter, D., EMC Corporation, U.S.

Yuen, H., Occupational hygienist & safety specialist, Canada

Wang, P. S., Harvard Medical School, U.S.

Weiss, K., Eddie Bauer Inc., U.S.

Wellness Works: Community Health Services Dept., County of Lambton, Canada

Workers’ Safety and Insurance Board (WSIB), Canada

Workplace Health & Safety Centre, Canada

Work Well, Canada

World Health Organization (WHO)

The Yukon Department of Energy, Mines and Resources, Canada
