# Supplementary material for: Are workplace health promotion programs effective at improving presenteeism in workers? a systematic review and best evidence synthesis of the literature
Source: BMC Public Health. 2011 May 26;11:395. doi: 10.1186/1471-2458-11-395 (PMC3123596; doi:10.1186/1471-2458-11-395)
Supplement: Additional file 3 — Quality Assessment Tool for Quantitative Studies. This file contains the Effective Public Health Practice Project's (EPHPP) tool used to assess the methodological quality of studies that passed relevance screening for inclusion in this review. [file 1471-2458-11-395-S3.PDF]

Additional File 3. Quality Assessment Tool for Quantitative Studies

COMPONENT RATINGS

A) SELECTION BIAS

(Q1) Are the individuals selected to participate in the study likely to be representative of the target population?

1 Very likely
2 Somewhat likely
3 Not likely
4 Can't tell

(Q2) What percentage of selected individuals agreed to participate?

1 80–100% agreement
2 60–79% agreement
3 less than 60% agreement
4 Not applicable
5 Can't tell

RATE THIS SECTION	STRONG	MODERATE	WEAK
See dictionary	1	2	3

B) STUDY DESIGN

Indicate the study design

6 Randomized controlled trial
7 Controlled clinical trial
8 Cohort analytic (two group pre + post)
9 Case-control
10 Cohort (one group pre + post (before and after))
11 Interrupted time series
12 Other specify ____________________________
13 Can't tell

Was the study described as randomized? If NO, go to Component C.
No Yes

If YES, was the method of randomization described? (See dictionary)
No Yes

If YES, was the method appropriate? (See dictionary)
No Yes

RATE THIS SECTION	STRONG	MODERATE	WEAK
See dictionary	1	2	3

C) CONFOUNDERS

(Q1) Were there important differences between groups prior to the intervention?

14 Yes
15 No
16 Can't tell

The following are examples of confounders:

17 Race
18 Sex
19 Marital status/family
20 Age
21 SES (income or class)
22 Education
23 Health status
24 Pre-intervention score on outcome measure

(Q2) If YES, indicate the percentage of relevant confounders that were controlled (either in the design (e.g. stratification, matching) or analysis).

25 80–100% (most)
26 60–79% (some)
27 Less than 60% (few or none)
28 Can't Tell

RATE THIS SECTION	STRONG	MODERATE	WEAK
See dictionary	1	2	3

D) BLINDING

(Q1) Was (were) the outcome assessor(s) aware of the intervention or exposure status of participants?

29 Yes
30 No
31 Can't tell

(Q2) Were the study participants aware of the research question?

32 Yes
33 No
34 Can't tell

RATE THIS SECTION	STRONG	MODERATE	WEAK
See dictionary	1	2	3

E) DATA COLLECTION METHODS

(Q1) Were data collection tools shown to be valid?

35 Yes
36 No
37 Can't tell

(Q2) Were data collection tools shown to be reliable?

38 Yes
39 No
40 Can't tell

RATE THIS SECTION	STRONG	MODERATE	WEAK
See dictionary	1	2	3

F) WITHDRAWALS AND DROP-OUTS

(Q1) Were withdrawals and drop-outs reported in terms of numbers and/or reasons per group?

41 Yes
42 No
43 Can't tell
44 Not Applicable (e.g., one time surveys or interviews)

(Q2) Indicate the percentage of participants completing the study. (If the percentage differs by groups, record the lowest).

45 80–100%
46 60–79%
47 less than 60%
48 Can't tell
49 Not Applicable (e.g., Retrospective case-control)

RATE THIS SECTION	STRONG	MODERATE	WEAK
See dictionary	1	2	3

G) INTERVENTION INTEGRITY

(Q1) What percentage of participants received the allocated intervention or exposure of interest?

50 80–100%
51 60–79%
52 less than 60%
53 Can't tell

(Q2) Was the consistency of the intervention measured?

54 Yes
55 No
56 Can't tell

(Q3) Is it likely that subjects received an unintended intervention (contamination or co-intervention) that may influence the results?

57 Yes
58 No
59 Can't tell

H) ANALYSES

(Q1) Indicate the unit of allocation (circle one)

community organization/institution practice/office individual

(Q2) Indicate the unit of analysis (circle one)

community organization/institution practice/office individual

(Q3) Are the statistical methods appropriate for the study design?

60 Yes
61 No
62 Can't tell

(Q4) Is the analysis performed by intervention allocation status (i.e., intention to treat) rather than the actual intervention received?

63 Yes
64 No
65 Can't tell

GLOBAL RATING

COMPONENT RATINGS

Please transcribe the information from the gray boxes on pages 1–4 onto this page. See dictionary for how to rate this section.

**A**	**SELECTION BIAS**	**STRONG**	**MODERATE**	**WEAK**	
		1	2	3	
**B**	**STUDY DESIGN**	**STRONG**	**MODERATE**	**WEAK**	
		1	2	3	
**C**	**CONFOUNDERS**	**STRONG**	**MODERATE**	**WEAK**	
		1	2	3	
**D**	**BLINDING**	**STRONG**	**MODERATE**	**WEAK**	
		1	2	3	
**E**	**DATA COLLECTION METHOD**	**STRONG**	**MODERATE**	**WEAK**	
		1	2	3	
**F**	**WITHDRAWALS AND DROPOUTS**	**STRONG**	**MODERATE**	**WEAK**	
		1	2	3	Not Applicable

GLOBAL RATING FOR THIS PAPER (circle one):

1	STRONG	(no WEAK ratings)
2	MODERATE	(one WEAK rating)
3	WEAK	(two or more WEAK ratings)

With both reviewers discussing the ratings:

Is there a discrepancy between the two reviewers with respect to the component (A-F) ratings?

No Yes

If yes, indicate the reason for the discrepancy

1 Oversight
2 Differences in interpretation of criteria
3 Differences in interpretation of study

Final decision of both reviewers (circle one):

1 STRONG
2 MODERATE
3 WEAK

Thomas, H., Ciliska, D., Dobbins, M., & Micucci, S. (2004). A process for systematically reviewing the literature: Providing the research evidence for public health nursing interventions. *Worldviews on Evidence-Based Nursing, 1*, 176-184.
