# Supplementary material for: Are workplace health promotion programs effective at improving presenteeism in workers? a systematic review and best evidence synthesis of the literature
Source: BMC Public Health. 2011 May 26;11:395. doi: 10.1186/1471-2458-11-395 (PMC3123596; doi:10.1186/1471-2458-11-395)
Supplement: Additional file 4 — Data Extraction Results for Included Studies Rated Strong. This file contains the data extraction results for the 4 studies included in this review that were rated as strong after being assessed for methodological quality. Data includes authors, date of publication, country, study design, setting, participants, interventions, outcome measurements, and key findings and limitations. [file 1471-2458-11-395-S4.PDF]

Additional File 4. Data Extraction Results for Included Studies Rated Strong (n = 4) [in alphabetical order]

**AUTHORS & COUNTRY**	**IJzelenberg et al. (2007), The Netherlands**
**STUDY DESIGN**	Cluster RCT & economic evaluation
**SETTING**	9 large companies (> 500 workers)
**PARTICIPANTS**	*Company inclusion criteria:* provide 2 clusters of workers with physically demanding jobs (e.g., warehouse operators), work ≥ 24 hrs/wk<br>*Intervention group:* 9 clusters, *n* = 258<br>*Control group:* 9 clusters, *n* = 231<br>*Characteristics:* mean age 41.3 yrs, 96-98% male<br>*Response rate:* : ~ 85%
**INTERVENTION**	*Intervention group:* integrative approach of 3 low back pain preventive measures - individually tailored education/training, treatment, & ergonomic advice<br>*Control group:* usual care by general physician or occupational physician according to Dutch guidelines<br>*Intervention period:* 12 months
**OUTCOMES MEASUREMENTS**	*Primary:* low back pain *(Nordic)*, sickness absence *(other questionnaire)*<br>*Secondary:* pain intensity *(Numerical Rating Scale)*, functional limitations *(Roland Morris)*, upper extremity complaints *(Nordic),* sickness absence & productivity losses *(other questionnaire)**, general health *(SF-12)*, quality of life (*EQ-5D)*<br>*Follow-up period:* 6 & 12 months
**KEY FINDINGS**	*Presenteeism improved?* No
**KEY LIMITATIONS**	Possible inaccurate outcome measures; small sample size; selective loss to follow-up; lack of contrast between 2 groups (due to mandatory occupational health care); participating companies more likely to have occupational health care; intervention not successfully implemented; individually tailored interventions not appropriate in team-based processes.

**AUTHORS & COUNTRY**	**Nurminen et al. (2002), Finland**
**STUDY DESIGN**	Multi-centred RCT
**SETTING**	Laundry company
**PARTICIPANTS**	*Inclusion criteria:* women in permanent, physically demanding work<br>*Exclusion criteria:* contraindications for physical activity; refused participation<br>*Intervention group:* *n* = 133<br>*Control group: n* = 127<br>*Characteristics:* females (*N* = 260), mean age 40 yrs<br>*Response rate:* 80%

**INTERVENTION**	*Intervention group:* worksite exercise 1 hr/wk for 8 months; 2 reinforcement sessions at 14 months<br>*Control group:* no intervention<br>*Intervention period:* 8 months
**OUTCOMES MEASUREMENTS**	Perceived work ability *(Work Ability Index, modified Nordic)**; sick leave<br>*Follow-up period:* 3, 8, 12, & 15 months
**KEY FINDINGS**	*Presenteeism improved?* Yes, only slightly at short-term follow up. At 12 months, work ability increased more in the intervention group than in the control (11%, 95% *CI* 0.2-21.9, $p = 0.04$).<br>No change in sick leave.
**KEY LIMITATIONS**	Awareness of intervention & baseline assessments could have led to ↑ exercise in control group; good perceived work ability at baseline.

**AUTHORS & COUNTRY**	**Takao et al. (2006), Japan**
**STUDY DESIGN**	RCT
**SETTING**	Sake brewery
**PARTICIPANTS**	*Inclusion/exclusion criteria:* none<br>*Characteristics:* 45 supervisors, mostly male, each has ~ 5 subordinates<br>*Intervention group:* $n = 150$, subordinates of 23 supervisors (mean age 50 yrs)<br>*Control group:* $n = 100$, subordinates of 22 supervisors (mean age 48.9 yrs)<br>*Response rate:* ~ 95%
**INTERVENTION**	*Intervention group:* supervisor education program regarding mental health promotion<br>*Control group:* waiting list<br>*Intervention period:* 3 months
**OUTCOMES MEASUREMENTS**	Subordinates' psychological distress *(Brief Job Stress questionnaire)* & job performance *(WHO-HPQ)**<br>*Follow-up period:* at end of 3-month intervention
**KEY FINDINGS**	*Presenteeism improved?* Yes, only for young male white-collar subordinates; significant improvement on job performance ($F = 5.40$, $p = 0.029$).
**KEY LIMITATIONS**	Inadequate randomization; intervention not blinded; small sample size; difficult for young supervisors to advise older subordinates; un-validated questionnaire in Japanese setting; only short-term effects evaluated.

**AUTHORS & COUNTRY**	**Von Thiele Schwarz et al. (2008), Sweden**
**STUDY DESIGN**	Cluster RCT
**SETTING**	6 workplaces of public dental care organization, ≥ 25 workers
**PARTICIPANTS**	*Characteristics:* female, mean age 46.6 yrs

	Intervention group : “physical exercise” group (PE), *n* = 62<br>*Intervention group :* “reduced-work hours” group (RWH), *n* = 50<br>*Reference group: n* = 65<br>*Response rate:* 99%
**INTERVENTION**	*PE intervention group:* 2.5 hrs/wk mandatory physical exercise, medium-to-high intensity<br>*RWH intervention group:* e.g., 37.5 hrs/wk (instead of 40 hrs/wk) for full-time workers<br>*Reference group:* no intervention<br>*Intervention period:* 6 months
**OUTCOMES MEASUREMENTS**	Biomarkers *(e.g., HDL, LDL, triglycerides, glucose, waist-to-hip ratio, blood pressure, heart rate)*; measures of physical activity, health-related factors, work ability *(other questionnaire)**; general & musculoskeletal symptoms *(Nordic)*<br>*Follow-up period:* 6 & 12 months after intervention
**KEY FINDINGS**	*Presenteeism improved?* No
**KEY LIMITATIONS**	High baseline physical activity for all groups; all groups increased physical activity levels; differences between workplaces; additional employees not recruited to compensate for reduced work hours, therefore increased work demands may have reduced potential beneficial intervention effects.

*Measure of presenteeism
