# Supplementary material for: Are workplace health promotion programs effective at improving presenteeism in workers? a systematic review and best evidence synthesis of the literature
Source: BMC Public Health. 2011 May 26;11:395. doi: 10.1186/1471-2458-11-395 (PMC3123596; doi:10.1186/1471-2458-11-395)
Supplement: Additional file 5 — Data Extraction Results for Included Studies Rated Moderate. This file contains the data extraction results for the 10 studies included in this review that were rated as moderate after being assessed for methodological quality. Data includes authors, date of publication, country, study design, setting, participants, interventions, outcome measurements, and key findings and limitations. [file 1471-2458-11-395-S5.PDF]

Additional File 5. Data Extraction Results for Included Studies Rated Moderate (n = 10) [in alphabetical order]

**AUTHORS & COUNTRY**	**Blangsted et al. (2008), Denmark**
**STUDY DESIGN**	Cluster RCT
**SETTING**	Office
**PARTICIPANTS**	N = 549 office workers<br>*Exclusion criteria:* hypertension, cardiovascular disease, symptomatic disc prolapses, severe neck disorders, neck & shoulder surgery, history of severe trauma, pregnancy<br>*Intervention group:* "Specific Resistance Training" group (SRT): *n* = 180, mean age 46 yrs, 70% female<br>*Intervention group:* "All-round Physical Exercise" group (APE): *n* = 187, mean age 43.9 yrs, 64.2% female<br>*Control group: n* = 182, mean age 44.9 yrs, 59.3% female<br>*Response rate:* ~ 40%
**INTERVENTION**	*SRT intervention group:* neck & shoulder resistance exercises 3x/wk<br>*APE intervention group:* encouraged to increase physical activity during work & leisure time<br>*Control group:* general health-promoting activities not including physical activity<br>*Intervention period:* 12 months
**OUTCOMES MEASUREMENTS**	Neck & shoulder musculoskeletal symptoms *(Nordic)*; perceived work ability *(Work Ability Index)**; sick leave<br>*Follow-up period:* end of 12- month intervention
**KEY FINDINGS**	*Presenteeism improved?* No
**KEY LIMITATIONS**	High Work Ability Index at baseline; insufficient program intensity; small sample size; high dropout & incomplete questionnaires; potential low intervention adherence; no neutral control.
**AUTHORS & COUNTRY**	**Block et al. (2008), USA**
**STUDY DESIGN**	RCT
**SETTING**	Kaiser Permanente of Northern California
**PARTICIPANTS**	*Inclusion/exclusion criteria:* all eligible<br>*Characteristics:* non-medical employees, *N* = 787, mean age 44 yrs, 74.3% female<br>*Response rate:* < 60%
**INTERVENTION**	*Intervention group: Alive!* (A Lifestyle Intervention Via Email):<br>to improve physical activity & diet behaviours<br>*Control group:* delayed control<br>*Intervention period:* 4 months

**OUTCOMES MEASUREMENTS**	*Primary:* diet & physical activity *(Health Risk Assessment)*<br>*Secondary:* health status & quality of life *(SF-8),*<br>presenteeism *(other questionnaire)**, Stage of Readiness for Change *(other questionnaire)*, self-efficacy *(other questionnaire)*<br>*Follow-up period:* end of 4-month intervention
**KEY FINDINGS**	*Presenteeism improved?* Yes; reduced difficulty in accomplishing work tasks in intervention group compared to control group (OR = 1.47, 95% CI 1.05-2.05, p = 0.02).<br>Intervention group also improved quality of life, health status, self-efficacy, stage of change.
**KEY LIMITATIONS**	Required internet access; possible spam-filtering of invitational messages/inactive email addresses; subjects that met diet & physical activity goals also included; possible low level of participation.

**AUTHORS & COUNTRY**	**Dababneh et al. (2001), USA**
**STUDY DESIGN**	Interrupted time series
**SETTING**	Meat-processing plant
**PARTICIPANTS**	Workers from 1 production line, N = 35<br>*Inclusion criteria:* all workers who handled filets & final products<br>*Exclusion criteria:* machine operators, line coordinator<br>*Characteristics:* mean age 39 yrs, 81% female<br>*Response rate:* 80 - 100%
**INTERVENTION**	36 min extra rest break time<br>2 different rest break schedules:<br>1. 12 3-min breaks<br>2. 4 9-min breaks<br>*Intervention period:* 6 wks total: 2 wks for each break schedule, separated by 1-wk interval
**OUTCOMES MEASUREMENTS**	Production rate *(video)**; discomfort in arms, neck, shoulders, back, legs *(other questionnaire)*; stress *(other questionnaire)*<br>*Follow-up period:* after each 2-wk rest break schedule
**KEY FINDINGS**	*Presenteeism improved?* Yes. Production rate of 4th period significantly higher (25-30%) than during baseline weeks with both schedules, [F (3, 12) = 22.49, p = 0.0].<br>9-min schedule preferred since it helped to improve lower extremity discomfort & well-being; taking more time for short breaks doesn't decrease production.
**KEY LIMITATIONS**	No attempt to counter balance order in which rest break conditions presented.

**AUTHORS & COUNTRY**	**De Boer et al. (2004), The Netherlands**

**STUDY DESIGN**	RCT
**SETTING**	Large electronic equipment manufacturing company
**PARTICIPANTS**	*Characteristics:* employees ≥ 50 yrs of age, mean age 53.4 yrs, 93% male<br>*Intervention group : n* = 61<br>*Control group: n* = 55<br>*Response rate:* 73%
**INTERVENTION**	Occupational health program for workers at risk for early retirement<br>*Intervention group:* assessment by occupational physician; detailed action plan; consultation with managers; referral to other specialists.<br>*Control group:* usual care<br>*Intervention period:* 6 months
**OUTCOMES MEASUREMENTS**	Early retirement, disability pension, work ability *(Work Ability Index)**, stress *(Utrechtse Burn-out Scale)*, quality of life *(Nottingham Health Profile)*, sick leave, satisfaction with occupational physician *(other questionnaire).*<br>*Follow-up period:* 6 months & 2 yrs after randomization
**KEY FINDINGS**	*Presenteeism improved?* Yes, at short-term follow up only (i.e., 6 months); intervention group had better work ability than control ($p < 0.001$).<br>Quality of life & stress also improved in intervention group.<br>At 2-yr follow-up: fewer workers in intervention group retired early but only to a small degree (85% in control vs. 72% in intervention).
**KEY LIMITATIONS**	None reported.

**AUTHORS & COUNTRY**	**de Kraker et al. (2008), The Netherlands**
**STUDY DESIGN**	RCT
**SETTING**	Dutch call centre
**PARTICIPANTS**	*Characteristics:* mean age 38 yrs, 73% female<br>*Intervention group : n* = 37<br>*Control group: n* = 23<br>*Response rate:* uncertain
**INTERVENTION**	*Intervention group:* used computer mouse with vibrating feedback signal to prevent hovering<br>*Control group:* used standard mouse without signal<br>*Intervention period:* 2 weeks
**OUTCOMES MEASUREMENTS**	Hovering behaviour *(The Observer software)*, productivity *(task performance, other questionnaire)**, usability & comfort *(other questionnaire)*, discomfort *(LEO: Dutch validated scale for body part discomfort).*<br>*Follow-up period:* end of 2-wk intervention
**KEY FINDINGS**	*Presenteeism improved?* No<br>Intervention group decreased their hovering time, but no differences found in productivity, discomfort, usability or comfort.

**KEY LIMITATIONS**	None reported.

**AUTHORS & COUNTRY**	**Mills et al. (2007), UK**
**STUDY DESIGN**	Quasi-experimental pre-post intervention-control study
**SETTING**	Office
**PARTICIPANTS**	Office-based service delivery employees<br>*Intervention group :* n = 519, 46% male, mean age 34.3 yrs<br>*Control group:* convenience sample, n = 1679, 54% male, mean age 34.5 yrs<br>*Response rate:* ~ 67%
**INTERVENTION**	*Intervention group:* Multi-component health promotion program<br>*Control group:* no intervention<br>*Intervention period:* 12 months
**OUTCOMES MEASUREMENTS**	Health risk factors *(Health Risk Assessment)*, absenteeism & work performance *(WHO-HPQ)**<br>*Follow-up period:* end of 12- month intervention
**KEY FINDINGS**	*Presenteeism improved?* Yes. Large mean increase in work performance scale of intervention group compared with control group, 0.79 (0.11), $p < 0.001$. Intervention group demonstrated greater improvement in all 3 outcomes.
**KEY LIMITATIONS**	Only 51% completed questionnaire at follow up; unknown reasons for dropping out; control group was recruited as “convenience” sample; possible non-equivalent groups.

**AUTHORS & COUNTRY**	**Rivilis et al. (2006), Canada**
**STUDY DESIGN**	Longitudinal quasi-experimental
**SETTING**	2 southern Ontario depots of large unionized courier company
**PARTICIPANTS**	*Intervention depot :* n = 71, mean age 40.4 yrs, 69% male<br>*Control depot:* n = 51, mean age 35.2 yrs, 68.6% male<br>*Response rate:* ~ 86%
**INTERVENTION**	*Intervention group:* Participatory ergonomic (PE) process<br>*Control group:* no intervention<br>*Intervention period:* 14 months
**OUTCOMES MEASUREMENTS**	Musculoskeletal disorder outcomes *(other questionnaire)*: perceived physical demands, organizational risk factors, pain; work function *(WLQ)**<br>*Follow-up period:* end of 14-month intervention
**KEY FINDINGS**	*Presenteeism improved?* Yes.<br>Intervention group: increased communication levels associated with increased

	work role function (WRF), *p* = 0.025; decreased pain post-intervention related to increased WRF, *p* = 0.049.
**KEY LIMITATIONS**	Some changes introduced late; short intervention and follow-up periods; barriers to implementation; low response rates; transfer between jobs.

**AUTHORS & COUNTRY**	**Tsutsumi et al. (2009), Japan**
**STUDY DESIGN**	Cluster RCT
**SETTING**	Medium-sized company producing electrical devices
**PARTICIPANTS**	Blue-collar workers<br>*Intervention group :* 6 assembly lines, *n* = 47 (42 general workers, 5 supervisors), 57% female, mean age 48 yrs<br>*Control group:* 5 lines, *n* = 50 (42 general workers, 8 supervisors), 36% female, mean age 44 yrs<br>*Exclusion criteria:* re-streamlining of lines (3 lines)<br>*Response rate:* 80 - 100%
**INTERVENTION**	*Intervention group:* participatory intervention focused on environment improvement/job redesign<br>*Control group:* no intervention<br>*Intervention period:* 12 months
**OUTCOMES MEASUREMENTS**	Mental health *(General Health Questionnaire, Job Content Questionnaire);* productivity *(WHO-HPQ)**<br>*Follow-up period:* 12 months
**KEY FINDINGS**	*Presenteeism improved?* Yes.<br>Intervention group: increased productivity *F* (4.05), *p* = 0.048; increased job control & supervisor/co-worker support; mental health scores remained the same.<br>Control group: decreased productivity & mental health scores.
**KEY LIMITATIONS**	Small sample size; more older workers lost at follow-up; psychometric properties of JCQ needs to be evaluated in Japan; potential information leakage in small factory; lack of longer follow-up data; possible burden of participatory approach on participants.

**AUTHORS & COUNTRY**	**Viola et al. (2008), UK**
**STUDY DESIGN**	Cross-over design
**SETTING**	Distribution company for electronic parts
**PARTICIPANTS**	94 white-collar workers on 2 floors; all invited to participate.<br>*1st floor:* *n* = 52, 26 female, mean age 34.9 yrs<br>*2nd floor:* *n* = 42, 19 female, mean age 37.4 yrs<br>*Response rate:* uncertain

**INTERVENTION**	Exposure to blue-enriched white light vs. white light during daytime work hours.<br>*Intervention period:* 8 wks total (4 wks under each type of light)
**OUTCOMES MEASUREMENTS**	Alertness, mood, sleep quality, performance *(Workplace Questionnaire)** mental effort, headache, eye strain, evening fatigue, irritability, concentration.<br>*Follow-up period:* end of each 4-wk intervention period
**KEY FINDINGS**	*Presenteeism improved?* Yes. Mean change for work performance from baseline score increased by 0.5 units, $p < 0.0001$, when exposed to blue-enriched white light.<br>Also improved vitality ($p = 0.0008$), activity ($p = 0.008$), energy ($p < 0.0001$), alertness ($p < 0.0008$), ability to concentrate ($p = 0.005$), ability to think clearly ($p < 0.0001$). Participants felt less tired ($p < 0.0001$).
**KEY LIMITATIONS**	Only self-rated outcome measures used.

**AUTHORS & COUNTRY**	**Wang et al. (2007), USA**
**STUDY DESIGN**	RCT
**SETTING**	16 large diverse companies (e.g., airline, insurance)
**PARTICIPANTS**	604 workers<br>*Characteristics: m*ean age 41 yrs, 70% female<br>*Intervention group : n* = 304<br>*Control group: n* = 300<br>*Response rate:* < 50% (uncertain)<br>*Inclusion criteria:* employees aged ≥ 18 yrs, covered by managed behavioural health plan, have at least moderate depression.<br>*Exclusion criteria:* lifetime bipolar disorder, substance disorder, recent mental health specialty care, suicidality.
**INTERVENTION**	*Intervention group:* structured depression outreach-treatment telephone program<br>*Control group:* usual care<br>*Intervention period:* 12 months
**OUTCOMES MEASUREMENTS**	Depression severity *(Quick Inventory of Depressive Symptomatology)*, work performance *(WHO- HPQ)**<br>*Follow-up period:* 6 & 12 months after intervention
**KEY FINDINGS**	*Presenteeism improved?* Yes.<br>Intervention group: Higher # effective weekly hours worked compared to control group at 6 months (β = 3.0, 95% *CI* 0.4-5.6, $p = 0.03$) & 12 months (β = 3.3, 95% *CI* 0.9-5.8, $p = 0.008$).<br>Effect on job performance not significant but consistently positive.
**KEY LIMITATIONS**	Screening tool may have misclassified cases; participants may have had differences in prevalence, severity, or impairment than nonparticipants; differences in subgroups & workforce characteristics.

*Measure of presenteeism
