# Supplementary material for: Are workplace health promotion programs effective at improving presenteeism in workers? a systematic review and best evidence synthesis of the literature
Source: BMC Public Health. 2011 May 26;11:395. doi: 10.1186/1471-2458-11-395 (PMC3123596; doi:10.1186/1471-2458-11-395)
Supplement: Additional file 6 — Description of Interventions Demonstrating Positive Effect on Presenteeism. This file contains detailed descriptions of the 10 interventions which demonstrated a positive effect on presenteeism. Data includes intervention goals, intervention delivery methods; and indicates whether subjects were screened, whether interventions were individually tailored, and whether incentives were used. [file 1471-2458-11-395-S6.PDF]

Additional File 6. Description of Interventions Demonstrating Positive Effect on Presenteeism (n = 10) [in alphabetical order]

STUDY	INTERVENTION	OTHER COMPONENTS
Block et al., 2008	*Alive!* (A Lifestyle Intervention via Email)<br>**GOAL:** To improve diet & physical activity (PA) through behaviour change.<br>• *Lifestyle & Barriers Questionnaires* used to tailor intervention.<br>• Participants chose 1 module to work on for 4 months: (a) ↑ PA, (b) ↑fruits & vegetables, or (c) ↓ fats & sugars.<br>• Received weekly messages of small-step goals to choose from; mid-week messages served as goal reminders.<br>• After choosing goal, taken to "personal home page": tips for achieving goals, addressing barriers, chat room, additional links, etc.<br>• Easy to use; tailoring based on *behaviour change model & stickiness* concept.	**DELIVERY METHOD:** email<br>**SUBJECTS SCREENED?** yes, via health risk assessment (HRA)<br>**TAILORED?** yes<br>**INCENTIVES?** no
Dababneh et al., 2001	Two different rest break schedules tested on same group: (i) 12 3-min breaks, (ii) 4 9-min breaks.<br>**GOAL:** To improve productivity & well-being.<br>• Received 36-min extra rest break time.<br>• 2 weeks per rest break schedule (separated by 1-wk interval).<br>• Could do whatever they liked during breaks.<br>• Longer breaks allowed workers to attend to their physical needs (e.g., restroom break) without interrupting group production.	**DELIVERY METHOD:** organization change<br>**SUBJECTS SCREENED?** no<br>**TAILORED?** no<br>**INCENTIVES?** no
de Boer et al., 2004	Occupational health program<br>**GOAL:** To improve work ability & quality of life; prevent early retirement.<br>• ≥ 3consultations between employee & occupational physician (OP).<br>• OP explored health, work-related, & social/psychological factors.<br>• Multidisciplinary: collaborated with supervisors/managers, GP, medical specialist, or psychologist.<br>• Involved changing work tasks, extra tools/aids, improvement in work relations, etc.	**DELIVERY METHOD:** workers' own OP, in collaboration with supervisors/managers<br>**SUBJECTS SCREENED?** yes<br>**TAILORED?** yes<br>**INCENTIVES?** no
Mills et al., 2007	Low-cost multi-component health promotion<br>**GOAL:** To ↓ health risks; ↑ work productivity & performance.<br>• 12 months<br>• Advice tailored to level of readiness to change health-related behaviours.<br>• Unlimited access to personalized health web	**DELIVERY METHOD:** email, paper packages, seminars<br>**SUBJECTS SCREENED?** yes (HRA)<br>**TAILORED?** yes<br>**INCENTIVES?** yes (lottery tickets)

	portal that included interactive online behaviour-change programs.<br>• Received tailored emails bimonthly on wellness topics relevant to them.<br>• Received 4 on-site seminars based upon 4 most prevalent health risks.	
Nurminen et al., 2002	Worksite exercise<br>**GOAL:** To improve work ability; ↓ sick leaves.<br>• 8 months<br>• Received personal prescription for exercise & counselling by PT, to ↑ self-directed PA during leisure time.<br>• 60-min group worksite exercise sessions 1x/wk.	**DELIVERY METHOD:** physiotherapist (PT), occupational health nurse (OHN)<br>**SUBJECTS SCREENED?** no<br>**TAILORED?** yes<br>**INCENTIVES?** no
Rivilis et al., 2006	Participatory ergonomic (PE) intervention<br>**GOAL:** To improve musculoskeletal (MSK) health.<br>• 14 months<br>• ECT had 4 training sessions re: ergonomic principles, assessments/measurements, identification & management of workplace risk factors.<br>• Iterative process; progressed according to PE Blueprint (i.e., identification, assessment, solution building & testing, implementation).<br>• 14 changes implemented (e.g. new tools, workstation modifications).<br>• Considered physical & psychosocial stressors.<br>• Changed work organizational factors (in addition to physical demands).<br>• Open forum for workers to voice concerns.	**DELIVERY METHOD:** *Ergonomic Change Team (ECT):* workers, unit manager, administrative assistant, human resource manager, corporate health & safety regional manager, ergonomic facilitator.<br>**SUBJECTS SCREENED?** yes<br>**TAILORED?** yes<br>**INCENTIVES?** no
Takao et al., 2006	Job stress education for supervisors<br>**GOAL:** To improve subordinates' psychological distress & job performance.<br>• 3 months<br>• Single-session, 60-min education program re: early awareness of mental problems, return-to-work support, subordinate consultations, work environment improvements, self-care recommendations.<br>• 120-min 'Active Listening Training' with role-playing.	**DELIVERY METHOD:** OP, psychologists<br>**SUBJECTS SCREENED?** no<br>**TAILORED?** no<br>**INCENTIVES?** no
Tsutsumi et al., 2009	Participatory intervention<br>**GOAL:** To improve mental health & job performance.<br>• 12 months<br>• Meetings held with human resource personnel (HR) to discuss work environment improvements.<br>• Facilitators (HR, factory chief) & supervisors	**DELIVERY METHOD:** participatory<br>**SUBJECTS SCREENED?** yes (stress surveillance, risk evaluation)<br>**TAILORED?** yes<br>**INCENTIVES?** no

	trained re: workplace mental health.<br>• Workers trained on hazard identification; used checklists to implement workplace improvements.<br>• Facilitators supported & sustained employee autonomous activities.<br>• Follow-up evaluations: to address barriers & suggest solutions<br>• Team-based, problem-solving approach based on employee involvement, shared work-related goals, & action planning.	
Viola et al., 2008	Blue-enriched white light<br>**GOAL:** To investigate its effects on job performance & alertness.<br>• 4 wks per lighting condition (white vs. blue light).<br>• Exposed during workday hours.	**DELIVERY METHOD:** workplace physical environmental change<br>**SUBJECTS SCREENED?** no<br>**TAILORED?** no<br>**INCENTIVES?** no
Wang et al., 2007	Structured depression outreach-treatment telephone program<br>**GOAL:** To evaluate effects on workplace outcomes.<br>• 12 months<br>• Care managers given 12 hrs of training; 60 min weekly supervision; caseload = 50-70 participants.<br>• For participants with significant depression, care managers recommended in-person psychotherapy& medication evaluation (via telephone).<br>• For those declining in-person treatment, care managers maintained regular telephone contact, frequency determined by need. Those with significant symptoms after 2 months were offered an 8-session cognitive behavioural psychotherapy program.<br>• All participants were mailed a psychoeducational workbook.<br>• Psychiatrist available to care managers if needed.	**DELIVERY METHOD:** licensed master's degree-level mental health clinicians (care managers)<br>**SUBJECTS SCREENED?** yes<br>**TAILORED?** yes<br>**INCENTIVES?** no
